# Pathway-Based Association Analyses Identified TRAIL Pathway for Osteoporotic Fractures

**DOI:** 10.1371/journal.pone.0021835

**Published:** 2011-07-08

**Authors:** Yin-Ping Zhang, Yao-Zhong Liu, Yan Guo, Xiao-Gang Liu, Xiang-Hong Xu, Yan-Fang Guo, Yuan Chen, Feng Zhang, Feng Pan, Xue-Zhen Zhu, Hong-Wen Deng

**Affiliations:** 1 The Key Laboratory of Environment and Genes Related to Diseases, Xi'an Jiaotong University College of Medicine, Ministry of Education, Xi'an, Shaanxi, People's Republic of China; 2 Department of Biostatistics, Tulane University School of Public Health and Tropical Medicine, New Orleans, Louisiana, United States of America; 3 The Key Laboratory of Biomedical Information Engineering, Xi'an Jiaotong University School of Life Science and Technology, Ministry of Education and Institute of Molecular Genetics, Xi'an, Shaanxi, People's Republic of China; 4 University of Shanghai for Science and Technology, Shanghai, People's Republic of China; Rutgers University, United States of America

## Abstract

**Introduction:**

Hip OF carries the highest morbidity and mortality. Previous studies revealed that individual genes/loci in the Tumor Necrosis Factor (TNF) -Related Apoptosis-Inducing Ligand (*TRAIL*) pathway were associated with bone metabolism. This study aims to verify the potential association between hip OF and TRAIL pathway.

**Methods:**

Using genome-wide genotype data from Affymetrix 500 K SNP arrays, we performed novel pathway-based association analyses for hip OF in 700 elderly Chinese Han subjects (350 with hip OF and 350 healthy matched controls).

**Results:**

The TRAIL pathway achieved a significant *p* value (*p* = 0.01) for association with hip OF. Among the 38 genes in the TRAIL pathway, seven genes achieved nominally significant association with hip OF (*p*<0.05); the *TNFSF10* (*TRAIL*) gene obtained the most significant *p* value (*p* = 1.70×10^−4^). SNPs (*rs719126*, *rs6533015*, *rs9594738*, *rs1805034*, *rs11160706*) from five genes (*CFLAR*, *NFKB1*, *TNFSF11*, *TNFRSF11A*, *TRAF3*) of the pathway had minor alleles that appear to be protective to hip OF. SNPs (*rs6445063* and *rs4259415*) from two genes (*TNFSF10* and *TNFRSF10B*) of the pathway had minor alleles (A) that are associated with an increased risk of hip OF, with the ORs (odds ratios) of 16.51 (95%CI:3.83–71.24) and 1.37 (95%CI:1.08–1.74), respectively.

**Conclusions:**

Our study supports the potential role of the TRAIL pathway in the pathogenesis of hip OF in Chinese Han population. Further functional study of this pathway will be pursued to determine the mechanism by which it confers risk to hip OF.

## Introduction

The prevalence of osteoporotic fractures (OF), especially hip OF, has now become a significant public health burden due to the associated high morbidity, mortality and tremendous health care cost [1], [2] The incidence of hip OF will reach 6.27 million worldwide with a resultant cost of >$34 billion by the year 2050 [1]. The major demographic change of hip OF will occur in Asia. In 1990, 26% of hip OF worldwide occurred in Asia, and this rate could rise to 37% by the year 2025 and to 45% by the year 2050 [2]. Genetic factors play an important role in susceptibility to hip OF [3], [4]. However, to date, the genetic determination of hip OF is still largely unknown.

A pathway-based association analysis approach is based on gene set enrichment analysis. The method ranks genes/SNPs genome-wide by the significance of association with a disease phenotype to generate a gene list, then uses a statistic enrichment score (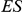) to examine whether a particular group of genes/SNPs within a certain functional pathway is enriched at the top (or bottom) of the list more than would be expected by chance [5]. This approach considers critical information about the interaction of a set of functionally related genes and their joint effects, and could potentially improve the power to detect genetic variants working in functional pathways. The method may play a major role in genetic analyses of complex diseases; for a pathway that contributes to a disease's risk, a single genetic variant within that pathway may have only limited contribution to the risk that might not be detectable (in regular genetic association analyses) if not otherwise be detected at the whole pathway level. Recently, through this new powerful approach, we have identified two novel pathways for wrist BMD and femur geometry in US whites [6], [7].

Previous studies revealed importance in the pathogenesis of bone metabolism for several individual genes/loci, or their transcripts and proteins in Tumor Necrosis Factor (TNF)-Related Apoptosis-Inducing Ligand (TRAIL) pathway, for example, *TRAIL*, *TRAILR1*, *TRAILR2*, *CFLAR*, *RANK*, and *OPG* [8], [9], [10]. However, it is unknown if the TRAIL pathway may contribute to osteoporotic fracture risk at the whole pathway level. We therefore undertook a novel, pathway-based association study to assess contribution of the pathway to hip OF risk.

## Materials and Methods

### Subjects

The study was approved by the institutional review board of Xi'an Jiaotong University. After signing an informed consent agreement, all subjects were assisted to complete a structured questionnaire including anthropometric variables, lifestyles, and medical history.

The sample consisted of 700 elderly Chinese Han subjects, including 350 with osteoporotic (low trauma) hip fractures (including 124 males and 226 females) and 350 controls (including 173 males and 177 females) (see Table 1 for the basic characteristics of these subjects). All the subjects were unrelated Chinese Han adults living in the city of Xi'an and its neighboring areas. Inclusion criteria for cases were (i) onset age >55 years, all female subjects were postmenopausal women; (ii) age <80 years to minimize the effect due to age, since previous studies showed that approximately half of females aged 80 years or older have fractures [11]; (iii) minimal or no trauma fractures, usually due to falls from standing height or less; (iv) fracture site at femoral neck or inter-trochanter regions; (v) hip fracture was identified/confirmed through diagnosis of orthopedic surgeons/radiologists according to radiological reports and X-rays. Patients with pathological fractures and high-impact fractures (such as due to motor vehicle accidents) were excluded.

**Table 1:** Characteristics of the study subjects.

	Case (n = 350)	Control (n = 350)
Age (years)	69.35 (7.41)	69.54 (6.09)
Weight (kg)	59.15 (12.05)	59.61 (10.84)
Height (cm)	162.84 (8.31)	159.41 (9.20)

Note: Data are shown as mean (standard deviation).

Healthy control subjects were selected from our established large database. They were geographically matched to the cases. Inclusion criteria for controls were: (i) age at exam >55 years, without any fracture histories (all female controls were postmenopausal); and (ii) not suffering from chronic diseases and conditions that might potentially affect bone mass, structure, or metabolism. The criteria may ensure that controls are less likely to suffer OF during their remaining life span compared with the general populations. The diseases/conditions mentioned above included chronic disorders involving vital organs (heart, lung, liver, kidney, brain), serious metabolic diseases (diabetes, hypo- and hyper-parathyroidism, hyperthyroidism, etc.), other skeletal diseases (Paget disease, osteogenesis imperfecta, rheumatoid arthritis, etc.); chronic use of drugs affecting bone metabolism (e.g., hormone replacement therapy, corticosteroid therapy, anti-convulsant drugs), and malnutrition conditions (such as chronic diarrhea, chronic ulcerative colitis). In addition, subjects taking anti-resorptive or bone anabolic agents/drugs, such as bisphosphonates were also excluded.

### Genotyping and quality control

Genomic DNA was extracted from peripheral blood leukocytes using a commercial isolation kit (Gentra systems, Minneapolis, MN, USA) following the standard protocols. SNP genotyping was performed using Affymetrix Human Mapping 500 K array set (Affymetrix, Santa Clara, CA, USA). Genotyping calls were determined from the fluorescent intensities using the dynamic model (DM) algorithm [12] as well as the B-RLMM (Bayesian Robust Linear Model with Mahalanobis Distance Classifier) algorithm [13]. DM calls were used for quality control of the genotyping experiment. BRLMM calls were used for the following pathway-based analyses. SNPs with a sample call rate <90%, with allele frequencies extremely deviating from Hardy-Weinberg equilibrium (*p*<10^−7^) and having a minor allele frequency (MAF) <0.01 in the total sample were discarded. The final SNP number for the analyses is 371,258. This SNP set covers 14,642 genes and yielded an average marker spacing of ∼7.9 kb throughout the human genome. Among these SNPs, 350 belong to the genes of the TRAIL pathway.

### Statistical Analysis

Statistical analyses on individual SNPs were first conducted by the Wald test implemented in Plink (version 1.03) [14] with age and sex as covariates. The main procedures of pathway-based analysis [15] are briefly summarized as follows:

1. Generation of gene-phenotype association rank and calculation of 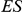: Among all the SNPs of a given gene G_i_, the SNP achieving the smallest *p* value in the single-marker association tests was used to represent the magnitude of association evidence of the gene. We ranked all genes by sorting their statistic values from the largest to smallest, denoted as a vector of the gene list L (r_1_,r_2_,…,r_N_). “N” equals to the total gene number contained in the GeneChip® Human Mapping 500 K set arrays. “r” represents the gene-phenotype association statistic value. Then, a weighted Kolmogorov-Smirnov–like running-sum enrichment score (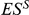) was calculated in Equation 1. For the given pathway S composed of N_S_ genes, by walking down the gene list L, we increased the running-sum statistic for the pathway when we encountered a gene in the S and decreased it when we encountered a gene not in the 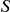.
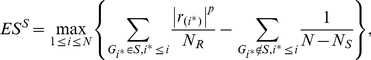
(1)Where 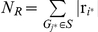 and parameter *p* (designated as 1 here) is designed to give higher weight to genes with extreme statistic values. The magnitude of the increment depends on the correlation of the gene with the phenotype. In short, 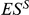 is the maximum deviation from zero encountered in the random walk. It will be high if the association signal is enriched at the top of list 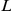, reflected by the significance level of observed 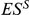 (i.e. nominal *p* value).
2. Permutation and nominal significance assessment: The phenotype data was shuffled and permutation (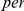) was done to compute a 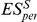 through repeating steps (1)∼(2) to estimate the nominal *p* value. A total of 1,000 permutations were done to create a histogram of the corresponding enrichment scores 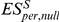 for the given pathway/gene set 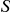. The nominal *p* value was estimated as the percentage of permutations whose 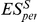 were greater than the observed 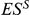.

To detect possible population stratification that may lead to spurious association results, we used Structure 2.2 software (http://pritch.bsd.uchicago.edu/software.html) to investigate the potential population substructure/stratification of the sample. The program uses a Markov chain Monte Carlo (MCMC) algorithm to cluster individuals into different cryptic sub-populations on the basis of multi-locus genotype data [16]. We performed the analysis assuming the number of population strata k = 2 and using 1,000 un-linked SNPs randomly selected genome-wide. To confirm the results achieved through Structure 2.2, we further tested population stratification in our sample using EIGENSOFT 2.0 software that uses both principal component analysis and a genomic control approach to correct possible statistical bias caused by ancestral differences [17], [18]. Reported *p* value was adjusted by the λ value estimated by genomic control method.

## Results

### Characteristics of study subjects

Basic characteristics of the 700 subjects are presented in Table 1. The STRUCTURE program revealed that all subjects in this Chinese sample were clustered together and could not be assigned into any subgroups, indicating that there was no significant population stratification within the sample. We also performed analysis using EIGENSOFT [17] and confirmed the homogeneity of the sample revealed by Structure; there is only one principal component that is significant in the principal component analysis (*p*<0.001), suggesting only one population ancestry existing for our sample. Finally, we used the genomic control method [18] to estimate the λ value, which equals to 1.02, suggesting again there is no population stratification in the sample.

### Pathway-based association analysis

The TRAIL pathway, annotated by the Ambion GeneAssist Pathway Atlas (Table 2), achieved a high NES of 1.76 with a *p* value of 0.01. Among the 38 genes in the TRAIL pathway, the gene with the most significant *p* value is *TNFSF10* (*TRAIL*, *p* = 1.70×10^−4^) (Table 3). Another six genes in the TRAIL pathway, which also contribute positively to the *ES* (i.e., the genes that ranked before or at the point of *ES*, also denoted as “leading edge genes”), are: *CFLAR* (*c-FLIP*, *FLIP*, *p* = 3.17×10^−3^), *TNFSF11* (*RANKL*, *p* = 6.06×10^−3^), *TNFRSF11A* (*RANK*, *p* = 7.63×10^−3^), *TNFRSF10B* (*TRAILR2*, *p* = 1.07×10^−2^), *TRAF3* (*p* = 1.67×10^−2^), and *NFKB1* (*NF-κB1*, *p* = 2.04×10^−2^). Among the 38 genes in the TRAIL pathway, 4 genes, *NFKBIE*, *IKBKG*, *RELB*, *DIABLO*, were not covered by Affymetrix 500 k Array Set, and the SNPs of the *XIAP* and *APAF1* genes were discarded for failure of passing the quality control criteria (Table 3).

**Table 2:** Component genes in the TRAIL pathway.

Gene Symbol	Gene ID	Genome Location	Full name	Protein Name
APAF1	317	12q23	apoptotic peptidase activating factor 1	APAF1
BID	637	22q11.1	BH3 interacting domain death agonist	BID
CBL	867	11q23.3	Cas-Br-M (murine) ecotropic retroviral transforming sequence	c-Cbl
REL	5966	2p13-p12	v-rel reticuloendotheliosis viral oncogene homolog (avian)	c-Rel
CASP3	836	4q34	caspase 3, apoptosis-related cysteine peptidase	Caspase3
CASP8	841	2q33-q34	caspase 8, apoptosis-related cysteine peptidase	Caspase8
CASP9	842	1p36.21	caspase 9, apoptosis-related cysteine peptidase	Caspase9
CYCS	54205	7p15.3	cytochrome c, somatic	CytoC
DAP3	7818	1q22	death associated protein 3	DAP3
TNFRSF10C	8794	8p22-p21	tumor necrosis factor receptor superfamily, member 10c, decoy without an intracellular domain	DCRI
TNFRSF10D	8793	8p21	tumor necrosis factor receptor superfamily, member 10d, decoy with truncated death domain	DCR2
FADD	8772	11q13.3	Fas (TNFRSF6)-associated via death domain	FADD
CFLAR	8837	2q33-q34	CASP8 and FADD-like apoptosis regulator	c-FLIP, FLIP
NFKBIA	4792	14q13	nuclear factor of kappa light polypeptide gene enhancer in B-cells inhibitor, alpha	I-KappaB-Alpha
NFKBIB	4793	19q13.1	nuclear factor of kappa light polypeptide gene enhancer in B-cells inhibitor, beta	I-KappaB-Beta
NFKBIE	4794	6p21.1	nuclear factor of kappa light polypeptide gene enhancer in B-cells inhibitor, epsilon	I-KappaB-Epsilon
CHUK	1147	10q24-q25	conserved helix-loop-helix ubiquitous kinase	IKK-Alpha
IKBKB	3551	8p11.2	inhibitor of kappa light polypeptide gene enhancer in B-cells, kinase beta	IKK-Beta

Gene Symbol	Gene ID	Genome Location	Full name	Protein Name
IKBKE	9641	1q32.1	inhibitor of kappa light polypeptide gene enhancer in B-cells, kinase epsilon	I-KK-Epsilon
IKBKG	8517	Xq28	inhibitor of kappa light polypeptide gene enhancer in B-cells, kinase gamma	I-KK-Gamma
NFKB1	4790	4q24	nuclear factor of kappa light polypeptide gene enhancer in B-cells 1	NF-KappaB1
NFKB2	4791	10q24	nuclear factor of kappa light polypeptide gene enhancer in B-cells 2 (p49/p100)	NF-KappaB2
TNFRSF11B	4982	8q24	tumor necrosis factor receptor superfamily, member 11b	OPG
TNFSF11	8600	13q14	tumor necrosis factor (ligand) superfamily, member 11	RANKL
PARP1	142	1q41-q42	poly (ADP-ribose) polymerase 1	PARP
TNFRSF11A	8792	18q22.1	tumor necrosis factor receptor superfamily, member 11a, NFKB activator	RANK
RELA	5970	11q13	v-rel reticuloendotheliosis viral oncogene homolog A (avian)	RelA
RELB	5971	19q13.32	v-rel reticuloendotheliosis viral oncogene homolog B	RelB
DIABLO	56616	12q24.31	diablo homolog (Drosophila)	SMAC
TRAF1	7185	9q33-q34	TNF receptor-associated factor 1	TRAF1
TRAF2	7186	9q34	TNF receptor-associated factor 2	TRAF2
TRAF3	7187	14q32.32	TNF receptor-associated factor 3	TRAF3
TRAF4	9618	17q11-q12	TNF receptor-associated factor 4	TRAF4
TRAF6	7189	11p12	TNF receptor-associated factor 6	TRAF6
TNFSF10	8743	3q26	tumor necrosis factor (ligand) superfamily, member 10	TRAIL
TNFRSF10A	8797	8p21	tumor necrosis factor receptor superfamily, member 10a	TRAILR1
TNFRSF10B	8795	8p22-p21	tumor necrosis factor receptor superfamily, member 10b	TRAILR2
XIAP	331	Xq25	X-linked inhibitor of apoptosis	XIAP

Note: (1) TRAIL, TNF-Related Apoptosis-Inducing Ligand.

(2) The Gene ID was retrieved from NCBI GenBank (http://www.ncbi.nlm.nih.gov/Genbank/).

**Table 3:** Association Results for the SNPs of Top Significance for Each Gene in the TRAIL Pathway.

Gene	SNP	Chr.	Physical location	Role	P value	Allele^a^	MAF^b^	OR (95%CI)^c^
APAF1	-	12	-	-	-	-	-	-
BID	rs424708	22	16588746	Downstream	0.07899	C	0.012	0.35 (0.11–1.13)
CBL	rs4938638	11	118580639	Downstream	0.3039	C	0.127	0.84 (0.60–1.17)
REL	rs842627	2	60994311	Downstream	0.08129	A	0.127	1.38 (0.96–1.99)
CASP3	rs4862379	4	185849707	Downstream	0.1001	C	0.081	1.40 (0.94–2.09)
CASP8	rs6723097	2	201954124	Intron	0.4147	C	0.468	0.91 (0.73–1.14)
CASP9	rs2042369	1	15715831	Intron	0.1818	A	0.039	0.66 (0.36–1.22)
CYCS	rs10239907	7	24905619	Upstream	0.1457	C	0.290	1.20 (0.94–1.53)
DAP3	rs821551	1	152501653	Intron	0.3871	A	0.180	0.88 (0.67–1.17)
TNFRSF10C	rs10111172	8	23025036	Intron	0.24	C	0.173	0.84 (0.64–1.12)
TNFRSF10D	rs4278155	8	23052724	Intron	0.4	C	0.441	0.91 (0.73–1.13)
FADD	rs11604809	11	69722984	Downstream	0.9726	C	0.466	1.00 (0.81–1.23)
**CFLAR**	**rs719126**	**2**	**201726075**	**Intron**	**0.00317**	**A**	**0.015**	**0.05 (0.01–0.36)**
NFKBIA	rs10132268	14	35017365	Upstream	0.1129	A	0.431	1.19 (0.96–1.46)
NFKBIB	rs2241704	19	44088175	Intron	0.9237	A	0.120	0.98 (0.71–1.37)
NFKBIE	-	6	-	-	-	-		-
CHUK	rs7909855	10	101957202	Intron	0.2635	C	0.460	0.88 (0.70–1.10)
IKBKB	rs10094577	8	42206733	Upstream	0.4423	C	0.013	0.68 (0.25–1.83)
IKBKE	rs2871360	1	203065616	Downstream	0.2916	C	0.428	0.89 (0.71–1.11)
IKBKG	-	X	-	-	-	-		-
**NFKB1**	**rs6533015**	**4**	**103712711**	**Upstream**	**0.02041**	**C**	**0.336**	**0.76 (0.60–0.96)**
NFKB2	rs11574851	10	104150949	Coding exon	0.4637	C	0.058	1.20 (0.74–1.96)
TNFRSF11B	rs7816831	8	119792244	Upstream	0.07085	C	0.201	1.29 (0.98–1.69)
**TNFSF11**	**rs9594738**	**13**	**41850145**	**Upstream**	**0.00606**	**C**	**0.080**	**0.55 (0.36–0.84)**
PARP1	rs3219058	1	222879529	Intron	0.1564	C	0.318	0.85 (0.67–1.07)
**TNFRSF11A**	**rs1805034**	**18**	**58178221**	**Coding exon**	**0.00763**	**C**	**0.314**	**0.72 (0.57–0.92)**
RELA	rs1466462	11	65175940	Intron	0.7124	A	0.193	0.95 (0.72–1.25)
RELB	-	19	-	-	-	-		-
DIABLO	-	12	-	-	-	-		-
TRAF1	rs2416804	9	120755950	Intron	0.7757	C	0.472	1.03 (0.83–1.28)

Gene	SNP	Chr.	Physical location	Role	P value	Allele^a^	MAF^b^	OR (95%CI)^c^
TRAF2	rs2811757	9	137071341	Intron	0.4854	A	0.131	0.88 (0.61–1.26)
**TRAF3**	**rs11160706**	**14**	**102431489**	**Intron**	**0.01667**	**A**	**0.341**	**0.75 (0.60–0.95)**
TRAF4	rs4794836	17	24106837	Downstream	0.4349	A	0.253	1.10 (0.87–1.40)
TRAF6	rs2201845	11	36512834	Downstream	0.3038	C	0.040	0.74 (0.42–1.31)
**TNFSF10**	**rs6445063**	**3**	**173676334**	**Downstream**	**0.00017**	**A**	**0.024**	**16.51 (3.83–71.24)**
TNFRSF10A	rs6557634	8	23116201	Intron	0.1974	C	0.022	1.65 (0.77–3.51)
**TNFRSF10B**	**rs4259415**	**8**	**22957513**	**Intron**	**0.0107**	**A**	**0.298**	**1.37 (1.08–1.74)**
XIAP	-	X	-	-	-	-		-

Note: (1) The SNPs significantly associated with hip OF are shown in bold.

(2) ^a^Minor allele of each SNP.

(3) ^b^Minor allele frequency calculated in our sample.

(4) ^c^Per-allele effect size of the minor allele is expressed by odds ratio (OR) and the 95% confidence interval of OR.

For the top SNP of each gene, the effect size of the minor allele is expressed by odds ratio (OR) derived from logistic regression analyses. The OR value is interpreted in the standard manner. A value of 1.0 indicates no effect. A value greater than 1.0 indicates that the minor allele is associated with an increased hip OF risk, and a value less than 1.0 implies that the minor allele is associated with a decreased risk, hence may be protective. Among the 7 SNPs representing the 7 leading edge genes, 5 SNPs had the protective minor allele. They are: *rs719126* of the *CFLAR* gene (minor allele A, OR = 0.05, 95%CI: 0.01–0.36), *rs6533015* of the *NFKB1* gene (minor allele C,OR = 0.76, 95%CI: 0.60–0.96), *rs9594738* of the *TNFSF11* gene (minor allele C, OR = 0.55, 95%CI: 0.36–0.84), *rs1805034* of the *TNFRSF11A* gene (minor allele C, OR = 0.72, 95%CI: 0.57–0.92), and *rs11160706* of the *TRAF3* gene (minor allele A, OR = 0.75, 95%CI: 0.60–0.95). The other two SNPs, *rs6445063* in the *TNFSF10* (*TRAIL*) gene and *rs4259415* in the *TNFRSF10B* (*TRAILR2*) gene, had a minor allele “A” with OR values of 16.51 (95%CI: 3.83–71.24) and 1.37 (95%CI: 1.08–1.74), respectively. The minor alleles of these two SNPs are significantly associated with an increased risk of hip OF (*P*<0.05) (Table 3).

## Discussion

**Figure 1:**
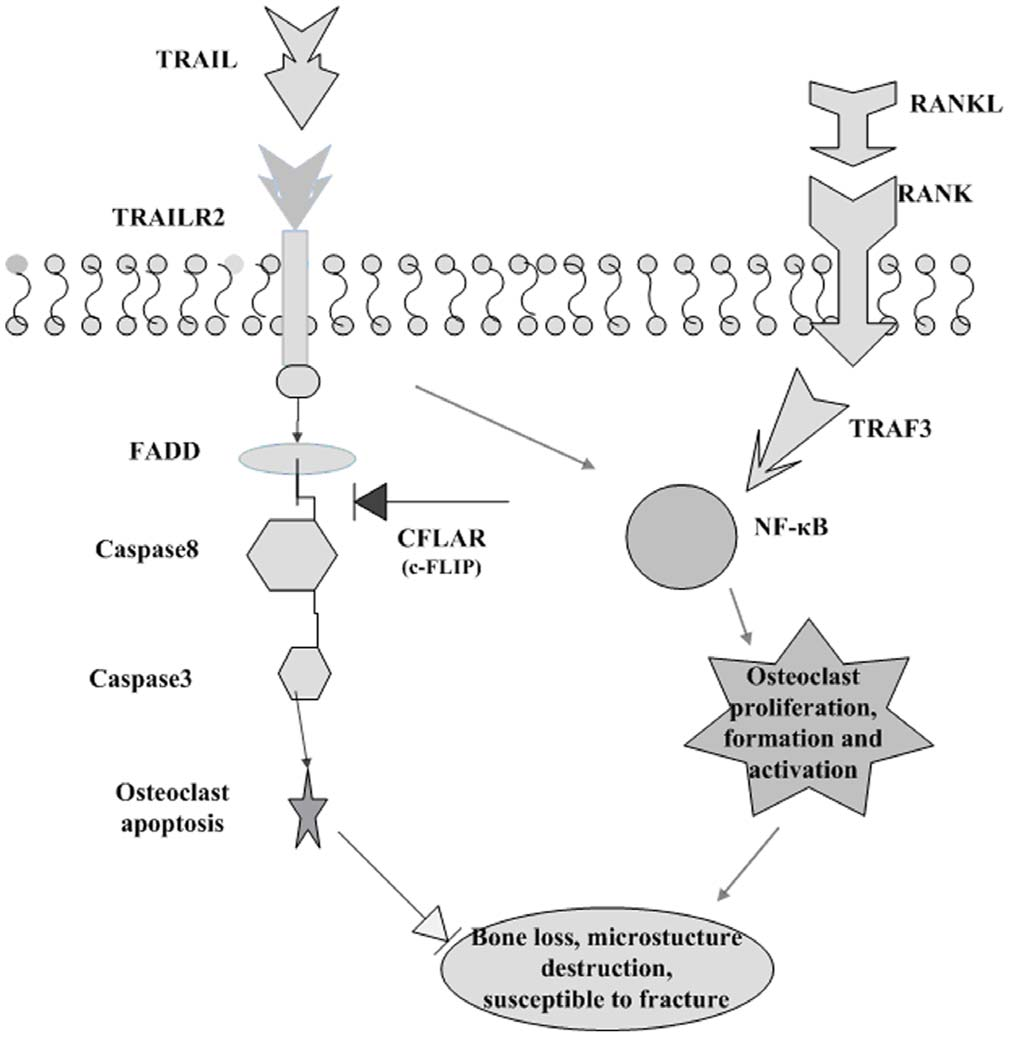
TRAIL pathway and the gene-gene interaction. The binding of TRAIL to TRAILR2 may induce the apoptosis of osteoclast. Increased c-FLIP levels may decrease the apoptosis of Osteoclast. The osteoclast apoptosis signal transduced by TRAIL/TRAILR2 was transformed to activate NF-κB. A key step in downstream signaling of RANKL/RANK is binding of TRAFs to RANK. Stimulation of RANK also results in strong NF-κB activation. NF-κB1(p50) is an important signal for osteoclast development and osteoclast function.

In this study, the TRAIL pathway was shown to be significantly associated with hip OF (*p* = 0.01) according to the pathway-based association analysis. This is the first time that the TRAIL pathway is implicated as an underlying factor for hip OF in Chinese. The pathway may contribute to bone mass variation via regulating osteoclast metabolism; previous findings have shown that the TRAIL pathway play roles in modulating the differentiation, function, survival and/or apoptosis of osteoclasts [19], [20], which may in turn accelerate bone resorption and consequently the susceptibility to hip OF. The core process of TRAIL and the functional interactions among the genes in the TRAIL pathway are depicted in Figure 1.

*TRAIL*, also known as *Apo2L*, is a widely recognized member of the tumor necrosis factor (TNF) ligand family [21], [22]. *TRAIL* is a typical type II membrane protein, binds to aggregates type I transmembrane receptors with cytoplasmic death domains (DD), which ultimately activate a protease cascade leading to apoptosis [23]. Interacting with five different receptors of the TNF receptor superfamily, *TRAILR1*, *TRAILR2*, *TRAILR3*, *TRAILR4* and *OPG*, the ligand is expressed constitutively in a wide range of tissues [22], [24], [25], including bone-related cells, such as osteoclast or osteoclast precursors. The binding of *TRAIL* to *TRAILR2* (DR5), which contains a conserved death domain (DD), may induce the apoptosis of osteoclast [19]. This may play an important role in the etiology of hip OF. *TRAIL* induces apoptosis in human differentiated osteoclasts by means of *TRAILR2*, and activates an intracellular pathway involving caspase-8 and Bid cleavage in the active forms [19]. However, in this study, SNP *rs6445063* from *TNFSF10 (TRAIL) gene* and SNP *rs4259415* from *TNFRSF10B* (*TRAILR2*) gene of the pathway had minor alleles (A) that are associated with an increased risk of hip OF, with the ORs (odds ratios) of 16.51 (95%CI:3.83–71.24) and 1.37 (95%CI:1.08–1.74), respectively. SNP *rs6445063* is located on chromosome 3q26.31 and has never been studied previously. SNP *rs4259415* is located on chromosome 8p21.3 and functions as intronic enhancer. According to the FASTSNP program (http://fastsnp.ibms.sinica.edu.tw), a change of “G→A” at *rs4259415* may lead to alter the binding sites for transcription factor from GATA-1 to CdxA and TATA, which may change the function of apoptosis related *TRAILR2* gene and accordingly alter the apoptosis of osteoclast. Furthermore, the apoptosis induced by death receptors can be modulated at several levels. Intracellular anti-apoptotic molecules can block the apoptotic signaling pathway. Soluble receptors inhibit TRAIL-mediated apoptosis in part by increasing baseline levels of CFLAR (c-FLIP), which competes with caspase-8 for binding to FADD, and inhibits active caspases [26]. Increased c-FLIP levels following exposure to soluble factors may decrease the apoptosis of osteoclast. Intracellular anti-apoptotic molecules can also divert the apoptosis signaling into alternative responses, i.e., the proliferation/survival of osteoclast via activating NF-κB [20]. Although lack of bone phenotype in mice with gene deletion of *TRAIL* was observed by Sedger et al [27], Yen et al [28] showed that *TRAIL* can induce osteoclast formation via direct engagement with the *TRAIL* death receptor. Yen et al [28], [29] suggested that TRAIL-induced osteoclastogenesisis was dependent on activation of NF-κB, ERK, and p38 MAP kinase and TRAF6 dependent. However *TRAF6* was not found to contribute positively to the ES as *TRAF3* in this study, suggests that there may be other factors that also can function in this capacity.

As the soluble decoy receptor of *TRAIL*, *OPG* was originally identified as a decoy receptor for *RANKL* [30] later found to be able to bind *TRAIL* [31]. *OPG* acts as antagonist for osteoclast apoptosis induced by *TRAIL*. But when *OPG* binds to the *RANKL* on the surface of osteoblast/bone matrix, it prevents *RANKL* from binding to its receptor, RANK, which inhibits the formation, activation and survival of multinucleated osteoclasts. In this process, the *RANKL/OPG* ratio is an important determinant of bone mass and skeletal integrity [32]. Up-regulation of the *RANKL* gene increases the RANKL/OPG ratio and enhances bone loss [32]. Our study found a significant association of SNPs (*rs9594738* and *rs1805034*) of *RANKL* and *RANK* with hip OF.

*NF-κB1* (p50) is a subunit of NF-κB transcriptional factor complex. The balance of survival (anti-apoptotic) and death (apoptotic) signals through NF-κB activation cascades results in normal bone homeostasis and healthy remodeling [33]. NF-κB activation through RANK/RANKL signal pathway is closely related with osteoporosis and the bone resorbing activity of osteoclasts [34]. After *RANKL* binds to *RANK*, a key step in downstream signaling is binding of TRAFs to specific sites of the cytoplasmic domain of *RANK* [20], [35], [36]. Stimulation of *RANK* results in strong *NF-κB* activation. *NF-κB1(p50)* is an important signal for osteoclast development and osteoclast function. Moreover, NF-κB can be activated in many signal cascades related to bone metabolism, e.g., NF-κB activation through the Fas/FasL system leads to enhanced osteoclastogenesis and reduced apoptosis, and it may also cause apoptotic cell death in osteoblasts [37].

Population stratification is an important source of spurious association in genetic association studies [38], [39]. However, these factors are unlikely to exist in our sample to interfere with our pathway-based association results. Our cohort came from an apparently homogenous Chinese north-west Han ethnicity population, living in Xi'an city and its neighboring areas. More importantly, in the analyses using Structure 2.2 [40], all subjects used in our study consistently clustered together as a single group, suggesting no significant population substructure. In the analysis using EIGENSTRAT [17], the measure for population stratification, λ, was very close to 1, which also suggests no stratification in our cohort. For the above reasons, our association results are unlikely to be plagued by spurious associations due to population admixture/stratification.

In summary, we applied a pathway-based analysis method to identify functional gene sets associated with hip OF. The significant enrichment of the TRAIL pathway genes among the top ranking genes associated with hip OF, together with the pathway's functional relevance to bone metabolism, strongly supports the important role of TRAIL in human hip OF risk. Further detailed and specific functional studies of the TRAIL pathway will be pursued to provide new insights into the etiology of hip OF.
